# The Cause and Treatment of Puerperal Sepsis

**Published:** 1900-11

**Authors:** F. B. Moore

**Affiliations:** Brushy Creek, Anderson Co., Texas


For Texas Medical Journal.

### The Cause and Treatment of Puerperal Sepsis.

BY F. B. MOORE, M. D., BRUSHY CREEK, ANDERSON CO., TEXAS.

The subject assigned me by our program committee is to me a most difficult one. Puerperal sepsis is, indeed, a broad field to be covered by one short paper, especially if we undertake to discuss all or all the morbid conditions of the puerperium due to septic infections. However, the committee lightened the task by confining me to "cause and treatment," rather than to the etiology, symptomatology, pathologic anatomy, history, etc., for which favor I extend my thanks.

Confining myself strictly to the cause of puerperal sepsis, I lay down this statement as the basis of what follows:

That the parturient canal, its walls, secretions and appendages constitute the *media* by which septic infection reaches the systemic circulations, and their power felt and the micro-organisms liberated or placed in proper culture soil for their propagation and spread. I admit that there are other septic conditions which complicate the puerperium, which have their origin other than through the parturient canal, but for reasons sufficient to myself I shall confine myself to those troubles which arise by and through the parturient canal.

Puerperal sepsis is the result of the entrance of micro-organisms from without into the canal or some of its appendages wherever they can find suitable lodgment that will serve as a proper culture bed for them. Tears, erosions, etc., act as doors which give these germs admittance into the system. I am persuaded that it is not impossible for a lying-in woman to have these micro-organisms within the parturient canal propuerperal, and thereby be the cause of septic infection *per se*. I grant this as possible, yet it is without question the exception and not the rule. To say that filth and uncleanliness will produce this condition will not do, for how many of us have waded through filth and dirt of the most dire and hurtful kinds and have to our astonishment seen both mother and child do well, with never a sign of sepsis, and yet to our olfactories there seemed to float on every particle of air not only micro-organisms, but ptominies and every form of putrifactive bacteria, both known and unknown.

I think it possible under all circumstances in puerperal sepsis to successfully trace the infections to some outside source, such as the attending physician, the midwife, nurse, or some unclean instrument used, as is often the case with midwives carrying a pewter syringe which was perhaps her grandmother's, which was never placed in hot water for fear it would lose its magic effect of keeping the patient in proper tense, or, as later practiced by some doctors, of

---

*Read at the Palestine meeting of the East Texas Medico-Chirurgical Association, July 5, 1900.

carrying a Davidson syringe with them on all occasions to be used for any and all purposes. These are but a few of the most common ways by which the infection is carried from place to place.

As to the micro-organism, which acts as the basis of this most dreadful malady, I believe that the streptococcus pyogenes is the one and one only which shapes and controls the morbid conditions of puerperal sepsis. While there are many others which are found associated in this trouble, some of which are strephelococcus tetanic bacillus, typhoid bacillus and bacillus communis coli, as well as the bacillus of scarlet fever and diphtheria, which I admit may become blended and produce mixed infections, which in some instances is most assuredly the cause of the fatality of these cases. But I maintain that for a true case of puerperal sepsis to exist streptococcus pyogenes must be present. My reason for admitting the possibility of mixed infections is that all micro-organisms that live and propagate at the normal temperature of the human body, reaching the proper culture bed will propagate regardless of other organisms which may be carrying on a similar process in the same or approximate tissues.

The treatment of puerperal sepsis consists, first, if possible, of removing the source of infection, the severity of which we can usually with safety judge by the range of temperature. If the temperature after delivering remains above 100° F. for twenty-four hours without evident cause it is safe to wash out the uterine cavity with hot water with or without some antiseptic added. Of these I prefer carbolic acid, 30 cc. to a quart of water, used in a fountain syringe, with Bozman's uterine douche. I usually make up two quarts of the solution, and after thoroughly cleansing the external genitals and vagina, I then attach the douche to pipe and invade the uterine canal clear up to fundus. This is best followed by a sufficient dose of sat. sal. sulph. magnes. given in tablespoonful doses every thirty minutes until bowels move freely. The hot water douche, and salts also, should be repeated often until convinced that severer measures are required. Should these fail to hold the temperature down this would be evidence sufficient to justify one in the use of the curette, which as to technique it is unnecessary for me to dwell, but will say that the danger to the uterine wall, and the bad results often following this operation when done by the most skilled operators, but reminds us that in these cases we can not be too careful with this useful but very dangerous instrument.

As to the constitutional treatment veratrum viride, salicyates soda and sulph. quinia. may be used with advantage; also saline purgatives, nutrient and tissue-making foods, alcoholic stimulants, hypodermoclysis of normal salt sal., and the use of anti-streptococcic serum. This last remedy is to my mind the coming treatment in all puerperal infections, not that I believe that it will eliminate all of the malady in mixed conditions, but it will eliminate one, and to my mind the most serious, of the micro-organisms and thereby reduce a complex pathological problem into one more simple.

